# Degradation Mechanism of Micro-Nanobubble Technology for Organic Pollutants in Aqueous Solutions

**DOI:** 10.3390/nano12152654

**Published:** 2022-08-02

**Authors:** Youbin Zhou, Dapeng Cao, Xianren Zhang

**Affiliations:** State Key Laboratory of Organic−Inorganic Composites, Beijing University of Chemical Technology, Beijing 100029, China; 2021200198@buct.edu.cn (Y.Z.); caodp@mail.buct.edu.cn (D.C.)

**Keywords:** nanobubbles, reactive molecular dynamics, sewage treatment, free radicals

## Abstract

Micro-nanobubbles (MNBs) technology has emerged as an effective means of sewage treatment, while the molecular mechanism for its pollutant degradation is still unknown. In this paper, the reactive molecular dynamics simulation technique is used to study the degradation mechanism of pollutants caused by shock-induced nanobubble collapse. We first demonstrate that the propagating shock wave can induce nanobubble collapse, and the collapsing nanobubble has the ability to focus mechanical energy via the converging motion of liquid in the interior of the bubble, leading to the formation of a high-speed jet with a much higher energy density. We also unveil the mechanical nature of long-chain pollutant degradation and the mechanism of free radical generation. Due to the impacting jet, the high-gradient flow has the ability to stretch the long-chain molecule and cause mechanical scission of the molecule in a homolytic manner. Finally, our simulation results reveal that adding ozone molecules to the collapsing bubble would introduce an additional dehydrogenation mechanism.

## 1. Introduction

Micro-nanobubbles (MNBs) technology has become an effective means of water treatment [1,2,3]. There are numerous advantages of using microbubbles and nanobubbles to control environmental pollution, including their large specific surface area, long existence time, self-pressurization effect, high mass transfer efficiency, high zeta potential at the interface, and spontaneous generation of free radicals [4,5,6,7,8,9,10,11,12,13,14,15,16]. The most common processes using MNBs technology for water treatment include flotation, aeration, disinfection, and advanced oxidation [2,3,17]. In particular, MNBs technique combined with reactive oxygen species has a wide application prospect due to their cheap, safe, and efficient characteristics [18]. The application of ozone MNBs to groundwater remediation demonstrated that ozone MNBs can greatly improve the remediation efficiency [19].

Most studies in MNBs technology focused on the generation methods, measuring techniques, and characterization of tiny bubbles. Limited efforts have been made on the mechanism of pollutant degradation. The accumulating evidence indicates that microbubbles and nanobubbles can be applied for water and wastewater treatment to improve the efficiency of chemical treatment, via catalyzing chemical reactions and enhancing the detoxification efficiency. However, the degradation mechanism has been rarely studied from the aspect of the molecular mechanism of how involved bubbles degrade the pollutants. The quantitative evaluation of the degradation mechanism is difficult, partially because of the complex nature of pollutant degradation, which involves the coupling of bubble oscillation and collapse with a variety of chemical reactions in pollutant degradation. It is very challenging to combine the two important aspects that occur in different time and space scales. We need to infer the underlying chemical mechanism involved in most cases and the transfer of electrons from the collective molecule motion of bubble collapse. However, the appearance of the reactive molecular dynamics (RMD) simulation technique [20,21,22,23] makes this kind of study possible now. The reactive molecular dynamics show a substantial advantage for dealing with this kind of question, and it is possible to directly observe the chemical events occurring inside a collapsing bubble with the atomistic resolution for long enough periods of time because the Reaxff force field has been developed by Duin et al. for the molecular dynamics simulation of reactive systems with chemical reactions involved [20,24,25,26].

The RMD method has been successfully used to study the effect of ultrasound-induced cavitation. The propagation of ultrasonic waves in liquids can lead to cavitation, in which the rapid collapse of microbubbles leads to local extreme conditions inside the bubbles. At the interface of water and glass, these kinds of local extreme conditions can initiate glass erosion [27]. With the Reaxff-RMD simulation, Nomura et al. investigated the shock-induced collapse of nanobubbles near the surface of amorphous silica. They showed that as the nanobubbles collapse, water jets form and collide with the silica surface to form hemispherical pits [28]. Vedadi et al. studied the collapse of carbon dioxide nanobubbles induced by high-speed shock waves and found that water molecules react with carbon dioxide molecules chemically [29]. For the long-standing suggestion that natural cavitation in primordial oceans was a dominant mechanism of organic molecule synthesis [30], Kalson et al. used RMD to directly demonstrate that bubble collapse may be an efficient energy source for the synthesis of biologically important molecules from pristine gases [31].

In this work, the ReaxFF-RMD simulation method is employed to study the chemical degradation of a typical pollutant taking place in a nanobubble collapse event of water treatment. As in most previous simulations, the shock waves are introduced to induce this violent bubble collapse. In reality, the shock waves can be caused by the instability of nanobubbles themselves or by the collapse of a larger nearby bubble [32]. We also consider the role of different gases inside the nanobubble in the degradation mechanism, in particular, the extensively used ozone in water treatment. Here, we choose NPnEOs as the model pollutants, which is a widely used nonionic surfactant [33], and has now become one of the most common pollutants.

## 2. Simulation Method and Details

In this work, we chose the reaction force field developed by Rahaman et al. [34] that includes C/H/O/N elemental parameters. This reaction force field has been successfully used in modeling the reaction of *·O* and *·OH* radicals with lipids [35], and also used in studying DNA damage caused by *·OH* radicals [36].

**Figure 1:**
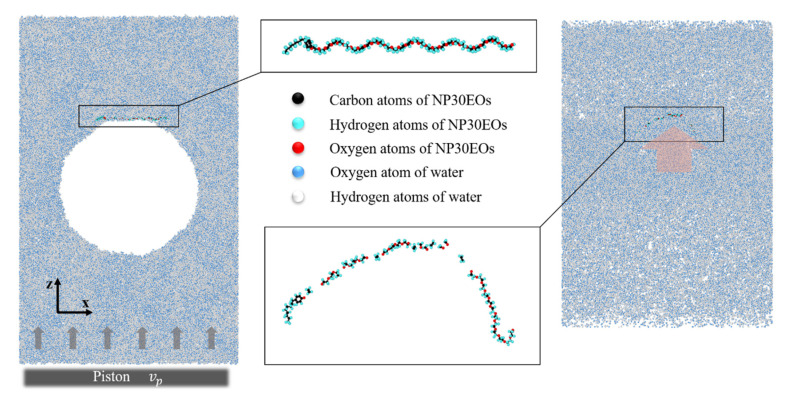
The initial configuration for a vacuum nanobubble system (**left**) and a typical configuration after the collapse of the nanobubble induced by the added shock wave (**right**). Magnified views of corresponding morphologies of the pollutant molecule, NP30EOs, are also shown (**middle**).

Our model consisted of three main components: the pollutant molecule NP30EOs, surrounding water, and gas molecules (oxygen or ozone) that were initially confined in the nanobubble (Figure 1). NPnEOs are nonionic surfactants, and their water solubility increases with the increase in the number of ethoxy groups (n). We selected NPnEOs containing 30 ethoxy groups and placed them at the upper end (along z-direction) of the nanobubble.

In order to save computing resources, we chose a pseudo-2D model for the simulation box. The size of the simulation box was set to 31.58 nm × 1.17 nm × 52.56 nm with 50,000 water molecules surrounding a vacuum bubble of 20 nm in diameter in the center of the box. We equilibrated the system for 50 ps under the NVT ensemble with T = 298 K (see Figure 1). In addition to the vacuum nanobubble, we also built the oxygen nanobubble and ozone nanobubble, respectively. First, we generated a smaller simulation box of 20 nm × 1 nm × 20 nm that only contained a number of ozone molecules. The ozone box was equilibrated for 50 ps under the NPT ensemble at T = 298 K and P = 101 kPa. After equilibration, a spherical part with a radius of 9.5 nm was excavated from the center of the box, which contained 37 ozone molecules. These ozone molecules were then embedded into the previous vacuum nanobubble of the large system to form the ozone-containing nanobubble. Using the same method, we also created an oxygen nanobubble containing 36 oxygen molecules.

Periodic boundary conditions were employed in the x and y directions, while the z directions aperiodic boundary condition was used. We placed a piston at the lower boundary of the simulation box (in the z-direction), which can compress the entire system at a velocity of *v_p_* within a rather short time. It is this short period of compression of the piston that achieves a shock wave. To prevent unreasonable contact of water molecules with aperiodic boundaries, we removed all the water molecules 1 nm away from the z-boundary. In this paper, we chose the compression velocity of the driving piston *v_p_* as 2 Km/s and the compression time as 3 ps.

With the simulation setup described above, we are ready to investigate the degradation of NP30EOs by the shock wave-induced collapse of nanobubbles containing different gases. All simulations were performed in LAMMPS [37] and visualization was implemented by Ovito. To account for the different distributions for liquid velocity, temperature, and liquid density, we divided the whole system into small squares with a perimeter of 1 nm in the x and y directions. Then, the dynamic characteristics of the bubble collapse can be monitored.

## 3. Results and Discussion

### 3.1. The Shock Wave-Induced Nanobubble Collapse and the Energy Focusing Effect

**Figure 2:**
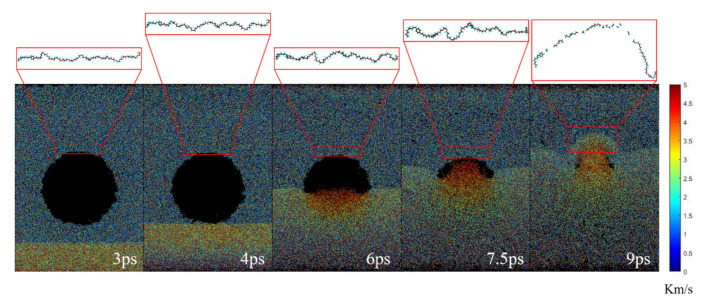
The time evolution of the vacuum nanobubble system under the action of exerted shock wave. The corresponding morphologies of the NP30EOs at the given simulation time are particularly shown on the top of each configuration.

**Figure 3:**
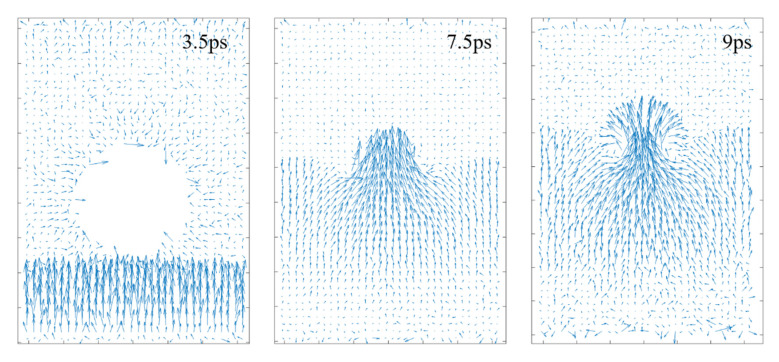
The distribution of liquid velocity at several simulation times, which correspond to, respectively, (**left**) the moment before the shock wave reaches the bubble, (**middle**) the jet forms, and (**right**) after the jet impacts the upper boundary of the bubble.

**Figure 4:**
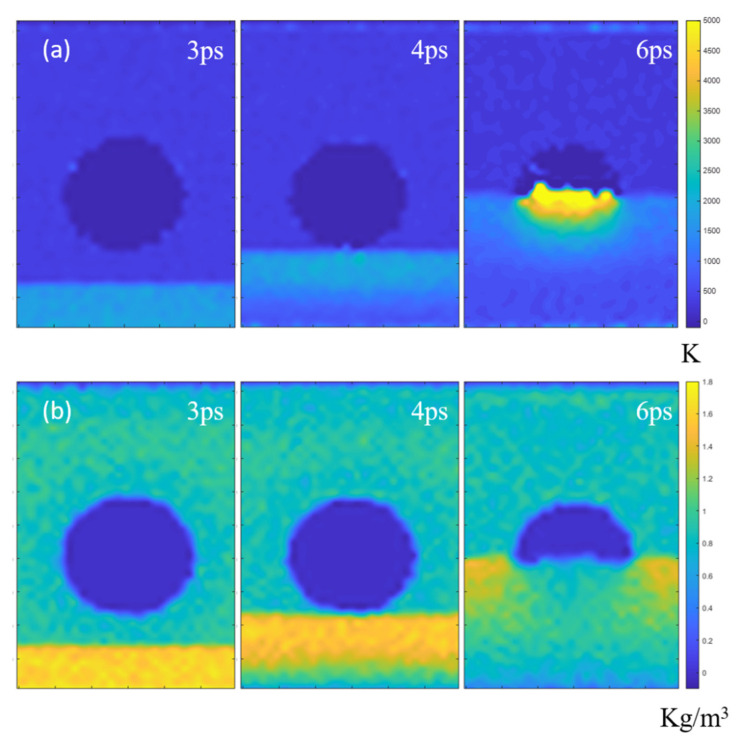
(**a**) The temperature contour and (**b**) the liquid density contour of the vacuum nanobubble system during a nanobubble collapse process.

**Figure 5:**
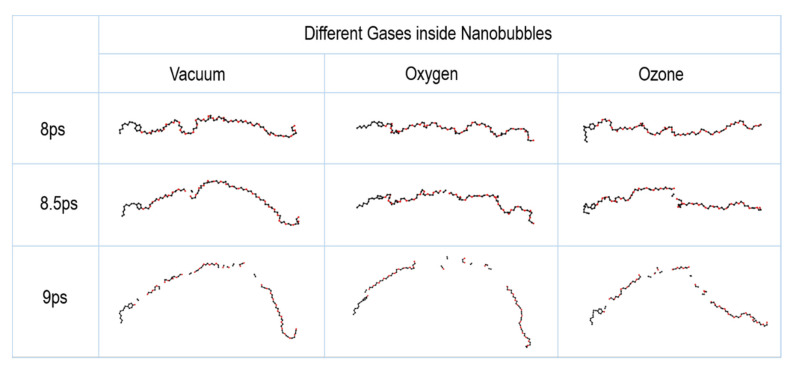
The morphology changes of the pollutant molecule and its fracture due to the collapse of different nanobubbles.

Our RMD simulations show that, as expected, the shock wave propagates inside the liquid, and after reaching the nanobubble, it induces the collapse of the nanobubble. The simulation results (Figure 2, Figure 3, Figure 4 and Figure 5) also reveal that the nanobubble can accumulate mechanical energy via the converging motion of liquid in the interior of the bubble. The focusing effect leads to the formation of a high-speed jet with a higher energy density, which would cause the degradation of nearby NP30EOs via covalent bond breakage.

Figure 2 shows typically the time evolution of a shock wave-induced collapse of the vacuum nanobubble, featured by the formation of a high-speed jet and the violent morphology change of the nearby NP30EOs. At 3 ps, the compression due to the driving piston stopped, and a high-speed and high-density area that initially formed at the lower system boundary began to propagate, indicating the generation of a shock wave. From 3ps to 4ps, the shock wave propagated upwards with a speed of ~2 km/s. At 4 ps, it reached the lower end of the nanobubble. Then, this propagation became no longer entirely upward due to the large difference in compressibility between the solvent and the bubble. As the shock wave continued to travel upward, the nanobubbles began to collapse. The liquid surrounding the bubble was pushed towards the center of the bubble, creating a high-velocity jet (see Figure 2 and Figure 3) which was also named as “water hammer (WH)” [38]. As clearly shown in Figure 3, although the shock wave velocity has an initial velocity of 2 km/s, the velocity of the formed jet can reach as large as 5 km/s. After that, the high-speed jet would have a strong collision with the upper wall of the bubble, and the jet would persist for a period of time after the collision (Figure 2 and Figure 3).

To interpret the accelerating effect, we also give the time evolution of liquid density in Figure 4b. When the system was subjected to the shock wave, a high-density area would be formed in the impacted area. This high-density region propagated forward in synchrony with the high-speed region. At the location where the front of the shock wave meets the bubble, the high density began to release due to the high compressibility of the bubble, but the surrounding area of the bubble is still in a high-density state. So, in order to release the excess density, the water molecules around the bubble are forced to move towards the center of the bubble. This tendency is confirmed by Figure 3, which also intuitively shows how water molecules move from the surrounding of the bubble to the bubble center. This kind of converging motion causes the acceleration of liquid in the bubble center and then the jet formation.

To more clearly illustrate the accelerating effect of liquid in the interior of nanobubbles and subsequent jet formation, we give the distribution of kinetic energy of liquid (Figure 4a). More specifically, we show the temperature distribution instead (Figure 4a), which is derived from kinetic energy by *T_K_* = [2/(3*N* × *k*)] × *K_E_* [39], where *K_E_* is the total kinetic energy of the molecular group, *T_K_* is the kinetic temperature, *N* is the number of atoms, and *k* is the Boltzmann constant. Here, the purpose of drawing the temperature cloud map is to display the kinetic energy of the molecules more intuitively. As shown in Figure 4a, the nanobubbles have the ability to focus their mechanical energy by generating jets with high energy density.

Figure 4a shows that at t = 6 ps, the collapse of the bubble produced an ultra-high temperature above 5000 K in the center of the bubble. This ultra-high temperature is caused by the high-velocity jet generated by the collapse of the nanobubble. Such high interior temperature is also predicted from the phenomenon of sonoluminescence, where the collapse of cavitation bubbles produces a momentary bright light. While emitting light, ultra-high temperatures (above 5000 K) are also predicted inside the bubbles [40,41,42,43].

Due to the small bubble size, the absolute value of the total energy for the collapse of a nanobubble is very small, but the energy density generated as a result of the converging motion in the bubble is enormous because of the focusing effect of the nanobubble. Consequently, the mechanical energy is accumulated into the very small volume of the nanobubble, and the violent collision of the interior flow with pollutants causes the bond breakages of the pollutants (Figure 2 and Figure 5). Therefore, nanobubbles provide an effective source of focusing mechanical energy that is capable of causing the specific degradation of long-chain pollutant molecules, as discussed below.

### 3.2. The Mechanical Nature of Long-Chain Pollutant Degradation and the Free Radical Production Due to Covalent Bond Breakage

Since the mechanical energy is accumulated into the very small volume of the nanobubble, covalent bond breakages of nearby long-chain pollutants (NP30EOs) take place (Figure 2 and Figure 5). Specifically, the bond breakage is caused by the mechanical forces that arise in the jet formation. When the impacting jet collides with the pollutant molecule, causing the bond breakage, various free radicals are observed.

**Figure 6:**
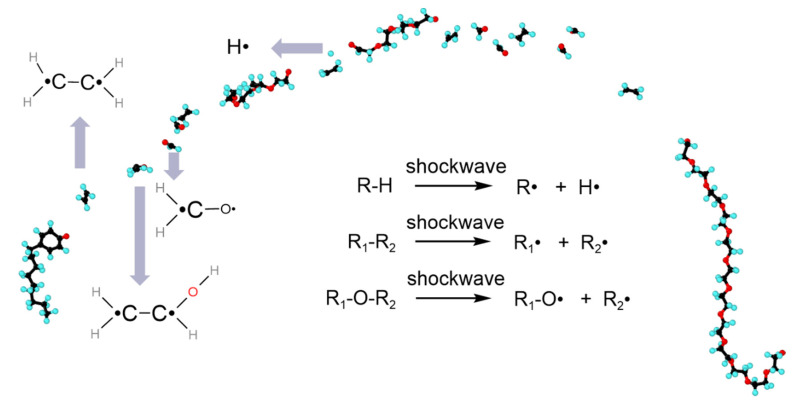
The rupture of NP30EOs after the collapse of the vacuum nanobubble and the generated radicals. In this figure, different reaction pathways for free radical generation are also given.

The mechanical nature of bond breakage is illustrated by the strong velocity gradients appearing in the liquid close to collapsing bubbles (Figure 3). Due to the impacting jet, a high-gradient flow is created in the surroundings of collapsing bubbles. The high-gradient flow has the ability to stretch the long-chain molecule at a high strain rate. When a pollutant molecule is caught within this gradient, the shear stress develops on the NP30EOs backbone. Under the action of the shear stress, the main chain of NP30EOs is stretched until bond scissions occur at sufficiently high force (Figure 2 and Figure 5). This is the reason why the middle of the main chain of NP30EOs is featured mostly with small molecular fragments, while the two ends are mostly fragments of large size (Figure 6).

Mechanical scission of long-chain molecules occurs in a homolytic manner and generates free radicals at the ends of the fragments [44]. When the NP30EOs molecule collides with the impacting jet, the bond breakage occurs in a homolytic manner, and various free radicals are observed. Figure 6 gives the typically generated free radicals, including *H*_2_·*C*−*C*·*H*_2_, *H*_2_·*C*−*C*·*HOH*, *H*_2_·*C*−*O*· and *H*· radicals. In this figure, we also summarize the reaction pathways for bond rupture. We stress here that for the collapse of the vacuum nanobubble, there was only one hydrogen atom that was found to be shed from the NP30EOs, generating a single hydrogen radical (Figure 6).

### 3.3. The Chemical Effect of Ozone Molecules and the Formation of Different Radicals

Above, we discussed the degradation of NP30EOs by the collapse of a vacuum nanobubble. We then discuss the effect of contained gas molecules on the pollutant degradation mechanism by considering the oxygen nanobubble and ozone nanobubble.

As shown in Figure 5, the addition of oxygen or ozone to the nanobubbles had a negligible effect on the cleavage of the main chain of NP30EOs, because the cleavage of the main chain of the pollutant molecule is mainly related to the speed of impacting jet, while the jet speed does not change significantly after adding ozone or oxygen. As shown below, the main effect of the type of gas content is reflected in the dehydrogenation reaction.

By observing the simulation results, we found that in the vacuum and oxygen nanobubble systems, only three or fewer hydrogen atoms were shed from the dissolved NP30EOs. In the nanobubble system with added ozone, about 15 hydrogen atoms were sloughed off from the NP30EOs. The difference indicates the chemical effect of the ozone in generating free radicals.

As shown in Figure 6, for the vacuum nanobubble system, independent simulation runs show that about 1–3 hydrogen atoms would be detached from the NP30EOs chain. Detailed inspection indicates that the shedding of hydrogen atoms is most frequently found in the center of the impacting jet with the strongest collision.

In contrast, the increased number of hydrogen atoms shed in the ozone nanobubble system is inseparable from the strong oxidizing property of ozone. There were two types of ozone oxidation: direct oxidation and indirect oxidation. The oxidative dehydrogenation here was caused by the indirect oxidation of ozone. Indirect oxidation is when ozone generates hydroxyl radicals in water, and the generated hydroxyl radicals play an oxidizing role.

**Figure 7:**
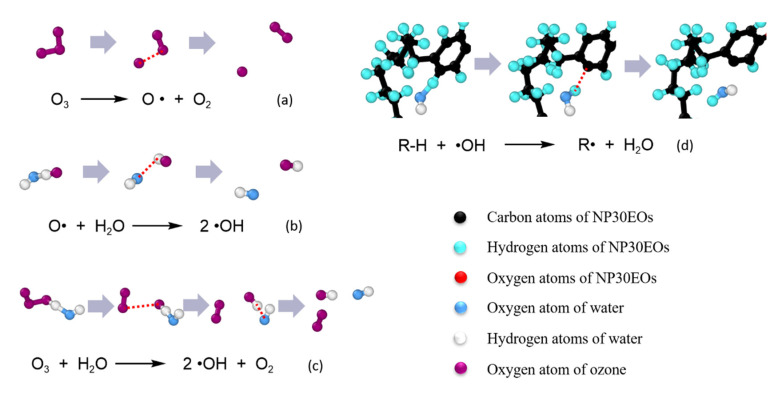
Additional chemical reactions taking place in the ozone nanobubble system. The red dotted line represents the bond to be broken in the given time step.

The effect of indirect ozone oxidation is given in Figure 7. As shown in Figure 7a, at the beginning of the simulation, a small fraction of ozone molecules in the nanobubble were decomposed into oxygen and oxygen radicals. The oxygen radicals decomposed by ozone would react with water molecules to generate two hydroxyl radicals (Figure 7b). After that, other ozone molecules would not continue to break down until the shock wave reaches the nanobubble. When the bubbles began to collapse, the jet first came into contact with the ozone molecules inside the nanobubbles, and the ozone molecules also reacted with water molecules under strong collisions to generate hydroxyl radicals, as shown in Figure 7c. Our simulation shows that all ozone molecules had been decomposed before the jet collided with NP30EOs. Therefore, there were a large number of hydroxyl radicals at the leading edge of the impacting jet. As shown in Figure 7d, these hydroxyl radicals would attack the hydrogen atoms on the NP30EOs, causing them to fall off. As a result, a large number of hydroxyl radicals at the front end of the jet would fully oxidize the NP30Eos molecule. This is also the reason for the fact that in the system with the addition of ozone molecules, the dehydrogenation of NP30EOs not only occurs in the middle part with the strong collision but also at both ends of the pollutant molecule. In summary, our simulation results indicate that the addition of ozone molecules would introduce an additional dehydrogenation mechanism, which is caused by hydroxyl radicals generated by the reaction of ozone with water.

## 4. Conclusions

In summary, we have used the reactive molecular dynamics simulation technique to systematically study the degradation mechanism of pollutants caused by the shock wave-induced nanobubble collapse. The simulation results indicate that the nanobubble can accumulate mechanical energy via the converging motion of liquid in the interior of the bubble, leading to the formation of a high-speed jet with a much higher energy density. Therefore, nanobubbles provide an effective source of focusing mechanical energy that is capable of causing the specific degradation of long-chain pollutant molecules. Due to the impacting jet, the high-gradient flow has the ability to stretch the long-chain molecule at a high strain rate and further cause bond scissions at sufficiently high stress, where mechanical scission of the pollutant molecules occurs in a homolytic manner and various free radicals were observed. Finally, we also revealed the additional chemical effect of ozone molecules in pollution degradation via strengthening the dehydrogenation reaction. After adding ozone molecules, the ozone molecules play a role of indirect oxidation, where it would generate hydroxyl radicals with water molecules, and then the hydroxyl radicals play a role in oxidative dehydrogenation of the pollutant molecule in water. Obviously, our simulation results indicate that the addition of ozone molecules would introduce an additional dehydrogenation mechanism. In short, this work provides a useful physical insight into the degradation mechanism of organic pollutes by using micro-nanobubbles technology, which would significantly speed up the development of micro-nanobubble technology in sewage treatment.

To the best of our knowledge, this paper is the first to try to apply the reactive molecular dynamics method to investigate the molecular mechanism of water treatment with the micro-nanobubble technology. Our paper demonstrates that RMD can provide a viable route in this regime, complementing experiments. For the elusive mechanisms of pollutant degradation via micro- and nano-bubbles, including the influence of factors such as acidity, alkalinity, and salts, the RMD approach can give unique insights into the microscopic aspects of related processes.

## Data Availability

The data that support the findings of this study are available from the corresponding author upon reasonable request.
